# Myelitis in a Young Patient With Testing Supportive for Both Angiostrongylus and Schistosoma Infection

**DOI:** 10.1177/19418744251370935

**Published:** 2025-08-20

**Authors:** Kristen Murray, Noriko Salamon, Doojin Kim, Michael G. Ho

**Affiliations:** 1 12222 David Geffen School of Medicine at UCLA, Los Angeles, CA, USA; 2 Department of Radiology, 12222 David Geffen School of Medicine at UCLA, Los Angeles, CA, USA; 3 Department of Neurology, 12222 David Geffen School of Medicine at UCLA, Los Angeles, CA, USA

**Keywords:** central nervous system infections, central nervous system parasitic infections, central nervous system infections, myelitis, transverse, spinal cord diseases, myelitis, central nervous system infections, infectious disease medicine, clinical specialty

## Abstract

Schistosoma and Angiostrongylus are both parasites which can cause central nervous system manifestations in humans. These parasites live in several overlapping geographic areas as well as share several clinical features. Diagnosis and treatment can be difficult given these infections are rare, have non-specific symptoms, and have definitive testing that takes days to weeks to result. We present a case of myelitis secondary to Angiostrongylus cantonensis infection with false-positive Schistosoma serologies complicating the diagnostic process in which the patient had an excellent clinical response to treatment with steroids and antiparasitic agents.

## Case Presentation

A twenty-three-year-old healthy male lifeguard native to Los Angeles developed a persistent mild headache 2 weeks after returning from Thailand (Bangkok, Chiang Mai, Koh Tau, and Kau Sak National Park) and the Philippines (Manila, Batangas, and Baler, Palawan). His primary care physician ordered an Magnetic Resonance Image (MRI) Brain that showed mild sinusitis and his headache improved with antibiotics. Several days later he developed burning pain over his left thigh, left abdomen, and left lower back leading to severe back muscle spasms and right-sided pain. A week later, he noticed a rash over the painful areas that resolved within a day. He was referred to an Infectious Disease specialist given his recent travel and was tested with CBC, Strongyloides Antibody (Ab), Trichinella Ab, Toxocara Ab, Schistosoma Ab, Anti-Nuclear Antibody, IgE, Stool parasite pathogen polymerase chain reaction, Stool Ova & Parasite, and Cyclospora/Cystoisopora.

About a week later, he developed unbearable back pain and presented to the emergency department. His pain had been migratory but now was most severe in the left shoulder and left upper back. He denied weakness, numbness, tingling, bowel or bladder incontinence, cough, night sweats, weight loss, or fever.

He had normal vital signs and was well-appearing without spinal tenderness or tenderness over the areas of pain. His neurological exam was benign: normal muscle bulk and tone, 5/5 muscle strength in the bilateral upper and lower extremities, 2+ reflexes, unremarkable sensory and cerebellar exam, and flexor plantar reflexes. Back examination revealed a red rash. Initial blood testing is listed in Table 1.

**Table 1.** Patient’s Initial Laboratory Values Upon Hospital Admission.

Component	Value	Reference Range Units
Complete blood count		
White blood cell count	5.69	4.16-9.95 × 10E3/uL
Red blood cell count	5.15	4.41 – 5.95 × 10E6/uL
Hemoglobin	15.5	13.5-17.1 g/dL
Hematocrit	45.5	38.5%–52%
Mean corpuscular volume	88.3	79.3 – 98.6 fL
Mean corpuscular hemoglobin	30.1	26.4-33.3 pg
MCH concentration	34.1	31.5-35.5 g/dL
Red cell distribution Width-SD	37.0	36.9-48.3 fL
Red cell distribution Width-CV	11.6	11.1%–15.5%
Platelet count, auto	246	143-398 × 10E3/uL
Mean platelet volume	10.3	9.3-13.0 fL
Nucleated RBC%, automated	0.0	%
Absolute nucleated RBC count	0.00	0.00-0.00 × 10E3/uL
Neutrophil percent	54.2	%
Lymphocyte percent	27.9	%
Monocyte percent	10.2	%
Eosinophil percent	6.5	%
Basophil percent	0.7	%
Immature granulocyte	0.5	%
Basic metabolic panel		
Sodium	140	135-146 mmol/L
Potassium	3.9	3.6 – 5.3 mmol/L
Chloride	100	96 – 106 mmol/L
Total CO_2_	25	20-30 mmol/L
Anion gap	15	8-19 mmol/L
Glucose	51	65-99 mg/dL
Creatinine	1.02	0.60-1.30 mg/dL
Estimated GFR	>89	mL/min/1.73 m^2^
Hematology, CSF		
CSF Appearance	Clear, colorless	-
Supernatant, CSF	Colorless	-
RBC count, CSF	1	0-10/cmm
WBC Count, CSF	53	0-5/cmm
Seg neutrophils, CSF	0	-
Lymphocyte, CSF	74	40%–80%
Monocyte, CSF	4	15%–45%
Eosinophil, CSF	22	0%–0%
Chemistry, CSF		
Glucose, CSF	57	43-73 mg/dL
Protein, CSF	67	15-45 mg/dL
Albumin IgG synth	4714	3500 – 5200 mg/dL
Albumin index	8.4	0.0-8.9
IgG index	0.6	0.0-0.6
Albumin CSF IgG synth	39.8	10-30 mg/dL
IgG serum IgG syn	920	700-1600 mg/dL
CNS IgG syn rate	<3.3	0.0 – 3.3 mg/24hr
IgG CSF	4.4	0.0-5.5 mg/dL
Oligoclonal bands, CSF	Positive	-
Oligoclonal bands number, CSF	2	0-1 bands
Viral, CSF		
Adenovirus Ab, CSF	<1:1	<1:1
Coxsackie A Ab type 2, CSF	<1:1	<1:1
Coxsackie A Ab type 4, CSF	<1:1	<1:1
Coxsackie A Ab type 7, CSF	<1:1	<1:1
Coxsackie A Ab type 9, CSF	<1:1	<1:1
Coxsackie A Ab type 10, CSF	<1:1	<1:1
Coxsackie A Ab type 16, CSF	<1:1	<1:1
Eastern equine IgG, CSF	<1:1	<1:2
Eastern equine IgM, CSF	<1:1	<1:2
Echo virus type 4, CSF	<1:1	<1:1
Echo virus type 7, CSF	<1:1	<1:1
Echo virus type 9, CSF	<1:1	<1:1
Echo virus type 11, CSF	<1:1	<1:1
Echo virus type 30, CSF	<1:1	<1:1
HSV 1 IgG index	0.03	<1:2
HSV 2 IgG index	0.02	<1.00
Influenza type A, CSF	<1:1	<1:2
Influenza type B, CSF	<1:1	<1.1
Measles (rubeola) IgG, IFA CSF	<1:64	<1:64
Measles (rubeola) IgM, IFA CSF	<1:1	<1:1
Measles interpretation, CSF	Antibody not detected	-
Mumps Ab IgG, IFA CSF	<1:8	<1:8
Mumps Ab IgM, IFA CSF	<1:1	<1.1
Mumps interpretation, CSF	Antibody not detected	-
St Louis IgM, CSF	<1:1	<1:1
St Louis IgG, CSF	<1:1	<1:1
Varicella-Zoster Ab CF, CSF	<1:2	<1:2
Western equine IgG, CSF	<1:1	<1:1
Western equine IgM, CSF	<1:1	<1:1
West nile virus, IgG	<1.30	<1.30
West nile virus IgG, CSF	0.05	<1.30
West nile virus, IgM	<0.90	<0.90
West nile virus IgM CSF	0.53	<0.90
Bacterial, CSF		
VDRL CSF	Nonreactive	Nonreactive

**Figure 1.**
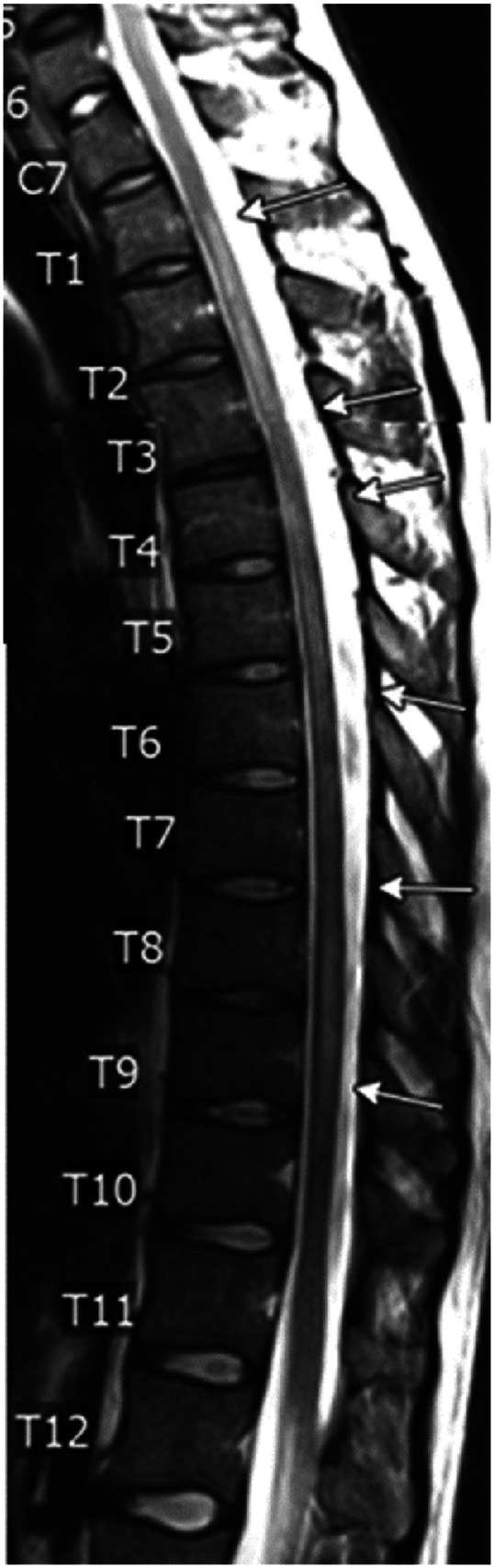
T2 weighted image of thoracic spine demonstrating multifocal segments of T2 hyperintensity at T1-3, T4-6, and T7-9 (image limited by motion artifact).

MRI thoracic spine without contrast showed multifocal segments of T2 hyperintensity at T1-T3, T4-T6, and T7-T9 as depicted in Figure 1.

Cerebrospinal Fluid (CSF) Studies were most notable for an elevated white blood cell count and an elevated eosinophil count. Full CSF studies are listed in Table 1. Given CSF eosinophilia, he had repeat CSF studies sent for additional infectious workup including samples sent to the National Institute of Health (NIH) and Center for Disease Control (CDC) for parasitic testing.

**Figure 2.**
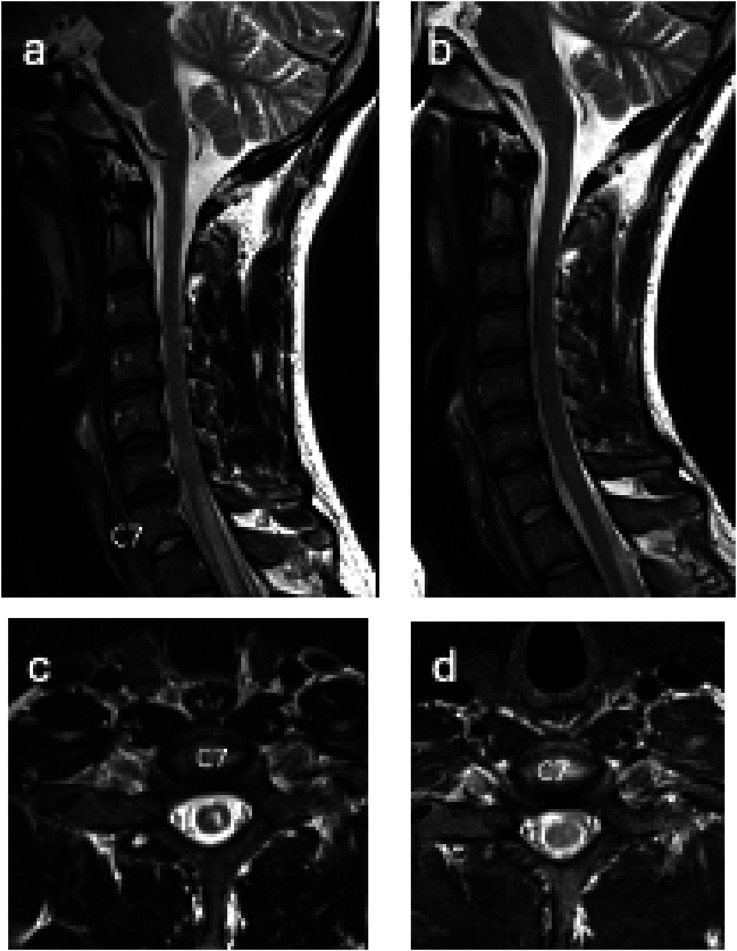
T2 weighted images of cervical spine before (A, C) and after treatment (B, D) demonstrating complete resolution of the T2 weighted signal abnormality within a central portion of the cord from the C7 level to below.

His pain and hypersensitivity progressed to his left elbow and forearm. Repeat MRI revealed extension of the thoracic abnormality now extending up to C6-7 level as seen in Figure 2A.

On hospital day 5, Schistosoma IgG antibody serology resulted positive leading to diagnosis of Neuroschistosomiasis. Stool O&P and multiple urine screens for Schistosoma were sent for confirmation, but all resulted negative which complicated the diagnosis. Neither molecular testing for Schistosoma species nor Schistosoma Antigen (Ag) testing was performed. At this time, his symptoms had started to resolve spontaneously. He was started on intravenous methylprednisolone for 5 days with complete resolution of his symptoms. He then completed a 5-day course of Praziquantal 30 mg/kg orally twice a day for definitive treatment of Schistosoma and a 3-month oral prednisone taper.

A few weeks later, qPCR results from CSF sent to the NIH and CDC resulted positive for *Angiostrongylus cantonensis*. His meningitis/ encephalitis antibody panel from the NIH and CDC was negative. Given concern for infection with both Schistosoma and Angiostrongylus vs Angiostrongylus with false positive Schistosoma serologies, he completed a 14-day course of Albendazole 400 mg orally twice a day. At 2-month follow up he had full resolution of symptoms and full resolution of transverse myelitis. (Figure 2)

## Discussion

### Schistosoma

Neuroschistosomiasis is a rare complication of Schistosoma infection and prognosis depends on early recognition and treatment. Schistosoma infections are generally asymptomatic or have mild rash, fever, chills, cough, and muscle aches.^1-3^ On rare occasions Schistosoma can spread to the central nervous system (CNS) and cause Neuroschistosomiasis, which typically presents with encephalitis if infected with *S. japonicum*, causing headache, seizure, and altered mental status, or myelitis if infected with *S. mansoni* or *S. haematobium* causing weakness, back pain, and urinary retention.^1-4^ With myelitis, spinal cord symptoms include lower limb weakness, bowel or bladder dysfunction, lower limb paresthesia, deep tendon reflex abnormalities, and sexual impotence in more than 80% of cases.^4^

Schistosoma typically occurs in Africa, the Arabian Peninsula, South America, China, Philippines, and Indonesia with various subspecies having predilection for different areas.^1,2^ Infection in humans occurs after contacting the parasite that lives in freshwater snails.^1,2^

The parasites mature over several weeks inside blood vessels. Some eggs travel to the bladder or intestines and are passed into urine or stool.^1-3^ Eggs can embolize to the CNS where they mature into adult forms.^3^ The eggs induce local eosinophilic inflammation causing damage, granuloma formation, and eventually fibrosis and demyelination.^1,3^

MRI spine may show signal hyperintensity on T2-weighted images, enlargement of the lower spinal cord and conus medullaris, thickened spinal roots, and a heterogeneous pattern of contrast enhancement on T1-weighted images.^4^

### Angiostrongylus

Angiostrongylus is the most common cause of eosinophilic meningitis and usually presents as transient meningitis.^5,6^ 90% of patients present with a headache and 40%–54% have paresthesias or hyperesthesia.^6,7^ Headache is often relieved by LP and patients with paresthesias may experience them for several week.^6-8^ Other common symptoms include fever, constipation, neck stiffness, nausea, vomiting, and transient abdominal pain with or without fever.^6-8^ Most patients have a self-limited course with complete recovery.^7^

Angiostrongylus primarily occurs in Southeast Asia with a predominance for Thailand and Malaysia.^9^ Infection in humans develops after eating infected raw or undercooked snails or slugs or from animals such as crab, freshwater shrimp, or centipedes.^5,9^

The worms migrate to the CNS, but do not produce eggs.^5^ Symptoms resolve as the worms die and the accompanying inflammation resolves. Treatment includes analgesics, corticosteroids, and CSF removal to relieve symptoms of increased intracranial pressure.^9,10^ There is debate about the benefit of antiparasitic agents.^9,10^

Laboratory findings include peripheral blood eosinophilia in approximately 60%–80% of patients.^4,11,12^ There is also generally a CSF eosinophilia that exceeds 10% in 95% of cases.^12^ CSF protein is usually elevated.^7^ MRI may demonstrate high signal intensities over the globus pallidus and cerebral peduncle on T1-weighted imaging, with multiple enhancing nodules in the brain and linear enhancement in the leptomeninges.^13^

### Clinical Decision Making

This case highlights the importance of considering a broad differential diagnosis in patients with atypical presentations of myelitis and eosinophilic CSF. This patient had a presentation that may be due to infection with Angiostrongylus and false positive Schistosoma serologies but could be from minimally symptomatic Schistosoma infection (likely without neurologic involvement) concurrent with Angiostrongylus infection. Early in his course, the clinical picture and positive Schistosoma serology seemed to fit the diagnosis of Neuroschistosomiasis, however the treatment team was unsatisfied with this diagnosis in isolation. In particular, the level of spinal cord involvement, cervico-thoracic rather than lumbosacral, was more suggestive of infection with *Angiostrongylus cantonensis* which often presents similarly. Additional factors such as travel to Southeast Asia, migratory pain, and rash also suggested Angiostrongylus as the primary causative agent and the diagnosis was confirmed with molecular testing. Despite the initial concern about Schistosoma, the lack of typical findings associated with Neuroschistosomiasis makes Angiostrongylus the more likely etiology for his myelitis, especially given Schistosoma serology can be falsely positive when cross reactive with other helminths including Angiostrongylus.^14^ He also had a negative stool O&P and negative urine screens for Schistosoma which continued to make the diagnosis of schistosomiasis less likely, however, given the treatment team was unable rule out this diagnosis completely, anti-parasitic treatment was prudent.

Patients often need to be treated without definitive testing given how long it takes to get results. Fortunately for him, treatment of both conditions with pulse dose steroids was indicated to decrease severity and length of symptoms and aid in recovery.^1,10,15^ Definitive therapy with antiparasitic agents for schistosomiasis is indicated after pulse dose steroids to reduce the risk of paradoxical reaction.^1,10^ The benefit of antiparasitic treatment for Angiostrongylus is currently debated and should be decided based on shared decision making with the patient.^15^ Ultimately, this case highlights the complexity of diagnosis of parasitic infections particularly when lab results are inconclusive or when cross-reactivity between different helminths complicates the interpretation. The clinical lesson here is the importance of evaluating all potential infectious causes, including co-infections, and being cautious of overreliance on a single serologic result when faced with an evolving clinical picture.

### Conclusion

Patients with demyelinating or inflammatory appearing CNS lesions, recent travel history, or atypical findings should be considered for workup for CNS helminths. Eosinophilic CSF Pleocytosis may further narrow the differential diagnosis for helminth infections, and a thorough travel history can help identify possible causative agents of concern. A single serologic test may be unreliable given possible cross reactivity, so a thorough diagnostic workup to evaluate for all potential etiologies, even with positive laboratory results for one pathogen, is important to evaluate and treat patients with an atypical presentation.

## Appendix

### Abbreviations

Ab: Antibody
Ag: Antigen
CDC: Center for Disease Control
CNS: Central Nervous System
CSF: Cerebrospinal Fluid
CT: Computed Tomography
LP: Lumbar Puncture
MRI: Magnetic Resonance Imaging
NIH: National Institute of Health
PCR: Polymerase Chain Reaction
